# Nutritional interventions to ameliorate the effect of endocrine disruptors on human reproductive health: A semi‐structured review from FIGO

**DOI:** 10.1002/ijgo.14126

**Published:** 2022-02-23

**Authors:** Gillian A. Corbett, Sadhbh Lee, Tracey J. Woodruff, Mark Hanson, Moshe Hod, Anne Marie Charlesworth, Linda Giudice, Jeanne Conry, Fionnuala M. McAuliffe, International Federation of Gynecology and Obstetrics (FIGO) Committee on Impact of Pregnancy on Long‐term Health and the FIGO Committee on Climate Change and Toxic Environmental Exposures

**Affiliations:** ^1^ UCD Perinatal Research Centre, UCD School of Medicine University College Dublin, National Maternity Hospital Dublin Ireland; ^2^ Program on Reproductive Health and Environment, Department of Obstetrics and Gynecology Philip R. Lee Institute for Health Policy Studies, University of California San Francisco California USA; ^3^ International Federation of Gynaecology and Obstetrics (FIGO) Committee on Impact of Pregnancy on Long‐term Health Dublin; ^4^ Institute of Developmental Sciences and NIHR Biomedical Research Centre, University of Southampton and NIHR Biomedical Research Centre University Hospital Southampton Southampton UK; ^5^ Mor Comprehensive Women’s Health Care Centre Tel Aviv Israel; ^6^ International Federation of Gynecology and Obstetrics (FIGO) Committee on Climate Change and Toxic Environmental Exposures; ^7^ Centre for Reproductive Sciences, Department of Obstetrics, Gynecology & Reproductive Sciences University of California San Francisco California USA; ^8^ Environmental Health and Leadership Foundation USA

**Keywords:** endocrine disruptors, lifestyle interventions, nutritional interventions, perinatal morbidity, reproductive outcomes

## Abstract

**Background:**

Endocrine disrupting chemicals have harmful effects on reproductive, perinatal, and obstetric outcomes.

**Objective:**

To analyze the evidence on nutritional interventions to reduce the negative effects of endocrine disruptors on reproductive, perinatal, and obstetric outcomes.

**Search strategy:**

A search of MEDLINE (PubMed), Allied Health Literature (CINAHL), EMBASE, Web of Science, and the Cochrane Database was conducted from inception to May 2021.

**Selection criteria:**

Experimental studies on human populations.

**Data collection and analysis:**

Data were collected from eligible studies. Risk of bias assessment was completed using the Cochrane risk of bias tool and the ROBINS‐I Tool.

**Results:**

Database searches yielded 15 362 articles. Removing 11 181 duplicates, 4181 articles underwent abstract screening, 26 articles were eligible for full manuscript review, and 16 met full inclusion criteria. Several interventions were found to be effective in reducing exposure to endocrine disruptors: avoidance of plastic containers, bottles, and packaging; avoidance of canned food/beverages; consumption of fresh and organic food; avoidance of fast/processed foods; and supplementation with vitamin C, iodine, and folic acid. There were some interventional studies examining therapies to improve clinical outcomes related to endocrine disruptors.

**Conclusion:**

Dietary alterations can reduce exposure to endocrine disruptors, with limited data on interventions to improve endocrine‐disruptor–related clinical outcomes. This review provides useful instruction to women, their families, healthcare providers, and regulatory bodies.

## Abstract 2

Nutritional interventions shown to reduce exposure to endocrine disruptors include avoidance of canned/processed or plastic‐packaged foods. Consumption of fresh/organic foods and vitamin C, iodine, and folic acid also reduce exposure.

## 1 INTRODUCTION

Endocrine disruptors are exogenous agents that interfere with synthesis, secretion, transport, metabolism, binding action, or elimination of natural blood‐borne hormones that are present in the body and are responsible for homeostasis, reproduction, and the developmental process.^1^ An important source of exposure to endocrine‐disrupting chemicals (EDCs) is diet, with ingestion being the dominant source^2^ of exposure globally. Dermal absorption and inhalation exposure to EDCs has also been reported. Building materials and industrial products also contain EDCs, including flame retardants and PVC.^3^, ^4^ Cosmetics, personal care products, anti‐microbial products and cleaning products, and household or industrial pesticides all contain EDCs.^5^

Endocrine disruptors affect a broad range of hormonal axes and physiological systems. Their effects have previously been divided into six types of toxicity including reproductive, developmental, and metabolic toxicity, neurotoxicity, immunotoxicity, respiratory toxicity, and carcinogenic effects.^6^ For a woman or couple considering a pregnancy, EDCs can affect conception, in‐utero development, neonatal life as well as long‐term child and maternal health. There are several windows of vulnerability throughout this process, where the woman and the fetus are particularly vulnerable to the adverse effects of endocrine disruptors. Oocyte integrity is compromised by EDCs, through oxidative stress and apoptosis^7^ and by reducing counts of antral follicles.^8^ This, too, has transgenerational effects. Pathological hormonal changes can affect gonadal development both in utero and after birth, causing estrogen‐mimetic effects on male gametogenesis with testicular failure, and testosterone‐related effects on female development, altering the onset of puberty and menarche and increasing the risk of precocious puberty.^9^ During the first weeks of fetal life, critical fetal neurogenesis requires adequate quantities of thyroid hormone. Where maternal exposure to EDCs interferes with thyroid function,^10^ dysfunctional neurogenesis can result.^2^ Some studies have linked prenatal endocrine exposure to cognitive and behavioral impairment, autistic spectrum disorders, and lower intelligent quotient (IQ) scores for the child in later life.^11^, ^12^, ^13^

The burden of morbidity with EDCs is complicated for many reasons: heterogeneity of exposure; multiple exposures at the same time; and difficulty in measuring the specific type of EDC exposure. EDCs can be either lipophilic or hydrophilic. Some lipophilic EDCs can accumulate in adipose tissue, giving these EDCs a relatively long half‐life in the human body. Other EDCs are hydrophilic and have shorter half‐lives though are often ubiquitously measured due to repeated and consistent exposure.^14^ Diabetogenic effects of EDCs are exerted through two main pathways. Alterations in fetal development of the pancreatic beta‐cells and the immune system cause type 1 diabetes mellitus later in life.^15^ Separately, endocrine disruptor interference with glucose and lipid metabolism contribute to insulin resistance and type 2 diabetes mellitus,^16^, ^17^ as well as metabolic toxicity and obesity.^18^, ^19^, ^20^ There is a synergistic effect here as some EDCs are themselves obesogenic, promoting weight gain, slowing weight loss, and increasing the volume of adipose and subsequent bioaccumulation of EDC.^21^ Endocrine disruptors have also been detected in the placenta and amniotic fluid, where they are proposed to be linked to adverse obstetric outcomes including preterm birth, gestational diabetes, pre‐eclampsia, and fetal growth restriction.^22^, ^23^, ^24^, ^25^

While there are strong bodies of evidence on the harmful effects of EDCs on female and male reproductive health and fetal development,^14^, ^26^ there is less clear guidance on how to minimize these effects. The pervasiveness of endocrine disruptors in every aspect of food cultivation, transport, storage, and preparation leads to exposure to women and their families. EDCs have the potential to have a life‐altering impact on families and societies in general and urgent global action is needed to reduce systematic exposure in every aspect of modern living. In the interim, the aim of the present systematic review was to summarize the evidence of nutritional interventions to minimize exposure to EDCs and to ameliorate the adverse effects of EDCs when exposure does occur. It is hoped that the present study will be a useful tool to women and their families, to healthcare providers, and to governmental bodies, empowering them in making evidence‐based decisions to reduce the effect of EDCs on the lives of their populations.

## 2 MATERIALS AND METHODOLOGY

In order to specify the study question, a Population‐Intervention‐Control‐Outcome (PICO) tool for the study was created through collaboration between the authors:

- **Population:** Human populations (both adults and children, male and female, and pregnant and non‐pregnant cohorts) with exposure to endocrine disrupting chemicals
- **Intervention:** Any nutritional intervention undertaken with the aim of reducing the exposure or effect of EDCs upon the population
  - Altering diet content: fruit/vegetables, organic foods, variety, avoidance of processed foods, Dietary Approaches to Stop Hypertension (DASH) diet, Mediterranean diet, high fiber diet, high protein diet, anti‐oxidant diet
  - Minimizing exposure to EDCs: avoidance of pesticides, limiting exposure to phthalates, breastfeeding, avoidance of processed baby food/canned foods, altering methods of food preparation
  - Iodine replacement
  - Folic acid replacement
  - Nutraceuticals: dietary supplementations including anti‐oxidant supplements (vitamin C, vitamin E, selenium, melatonin)
  - Microbiome: probiotic therapy or correction of dysbiosis
- **Control:** Placebo or non‐exposure to the intervention
- **Outcomes:** Centered around reproductive, maternal, and fetal health
  - Fertility: pregnancy rate, both male and female. Fertility parameters and assisted reproduction
  - Pregnancy outcomes: placental bioaccumulation, miscarriage, pregnancy loss, adverse obstetric outcomes including preterm birth (PTB), pre‐eclampsia, gestational diabetes (GDM), and fetal growth restriction
  - Fetal development: neurocognitive morbidity linked to thyroid dysfunction, HPA axis alteration and steroidogenesis, congenital fetal anomaly
  - Maternal health: thyroid metabolism, insulin resistance, diabetes, obesity
  - Exposure to endocrine disruptors: typically measured as serum or urine levels of endocrine disruptor metabolites

The search was created using the PICO structure to create a concept map of Medical Subject Headings (MeSH) search terms. The search methodology is reported here using the Preferred Reporting Items for Systematic Reviews and Meta‐Analyses (PRISMA) guidelines.^27^

### 2.1 Eligibility criteria

For the search, inclusion and exclusion criteria were based on the study question and characteristics as described in the PICO above.

Inclusion criteria were as follows: original research articles; observational cohort studies – both retrospective and prospective; experimental studies – both prospective and non‐randomized control/crossover studies; human studies; and English or non‐English reporting. Exclusion criteria were as follows: non‐human subjects; reviews or other non‐original research articles; exposure or intervention not reported; and results not reported.

Studies that met the selection criteria were screened by title and abstract, filtering the results for articles aligned with the PICO. Articles with titles and abstracts in keeping with the aim of the review were then moved forward for full article review. Where it was unclear from title and abstract review if the article met the inclusion criteria, it too was included for full review.

### 2.2 Information sources

The *Cochrane Handbook for Systematic Reviews of Interventions* and the Preferred Reporting Items for Systematic Reviews were used to guide the systematic review. The following databases were searched: MEDLINE (PubMed) 1966–2021; Allied Health Literature (CINAHL) 1990–2021; EMBASE 2000–2021; Web of Science 2000–2021; and the Cochrane Database 1996–2021. The search was performed on February 27, 2021, and was repeated on May 15, 2021. Reference lists for included articles were hand‐searched for additional articles meeting the inclusion criteria.

### 2.3 Search strategy

All relevant studies were included regardless of language or publication status (published, unpublished, in press, or in progress). Reference lists for all articles yielded through the database search were examined for additional articles for inclusion, including published reviews on this topic or similar topic content. The PICO structure was used to create a concept map of MeSH search terms of the PICO. Each concept of the PICO was used as a concept title, and a comprehensive search of each concept was then created using the advanced search function on each database. The concepts were then combined using Boolean search terms (AND/OR) as below:

- Patient: “Endocrine Disruptor Exposure”
  AND
- “Pregnancy”
  OR
- Intervention: “Diet Content Intervention” OR “Minimising EDC Exposure” OR “Iodine Replacement” OR “Folic Acid Replacement” OR “Neutraceuticals” OR “Microbiome”
  OR
- Outcomes: “Fertility” OR “Pregnancy Outcomes” OR “Fetal Development” or “Maternal Health”

Search filters were also used, based around the inclusion and exclusion criteria. Filters included randomized controlled trials, clinical trials and human‐only studies. All search results were then collated into one master search results folder on reference management software, EndNote.^28^

### 2.4 Selection process

Results of all database searches were collected on reference management software.^28^ Duplicates were removed and article title and abstracts were then screened for inclusion. Articles were screened by author GAC using titles and abstracts against the selection criteria. A second author (SL) screened 25% of search results independently, with no discrepancies between the two. During screening, if an abstract was deemed eligible or inconclusive, the full study article was then examined in close detail. Articles were then deemed suitable for inclusion thereafter.

### 2.5 Process of data collection

After an article was deemed eligible for inclusion by the two reviewers, the data were examined and manually collected by two reviewers (GAC, SL). Data were collected on a data collection sheet created for the present study. Where data appeared incomplete or additional information was required, the corresponding author of the study was contacted. The review was reported using the PRISMA guidelines.^27^

### 2.6 Data items

Target data included study author name, year and location of publication, study design, study population and size, and specific intervention and control group regimens. The outcome domains centered around the outcomes identified in the PICO: fertility; pregnancy outcomes; fetal development; maternal health; and changes in exposure to EDCs.

### 2.7 Study risk of bias assessment

For randomized controlled trials and crossover trials, the Risk of Bias and methodological quality was assessed using the Cochrane risk‐of‐bias tool version 6.2.^29^, ^30^ Methodological domains in this assessment include random sequence generation, allocation concealment, blinding of participants and personnel, blinding of outcome assessment, incomplete outcome data, selective reporting, and other biases. The ROBINS‐I tool was used to assess each domain of bias for non‐randomized trials. Domains of bias included confounding bias, selection bias, bias in classification of interventions, bias due to deviations from intended interventions, bias due to missing data, bias in measurement of outcomes and bias in selection of the reported results. Bias was judged by two reviewers (GAC, SL). We evaluated each of the domains separately as this is consistent with previous empirical analyses.^31^, ^32^, ^33^ If discrepancies occurred, the senior author (FMcA) was consulted to resolve assessments.

## 3 RESULTS

### 3.1 Study selection

**FIGURE 1:**
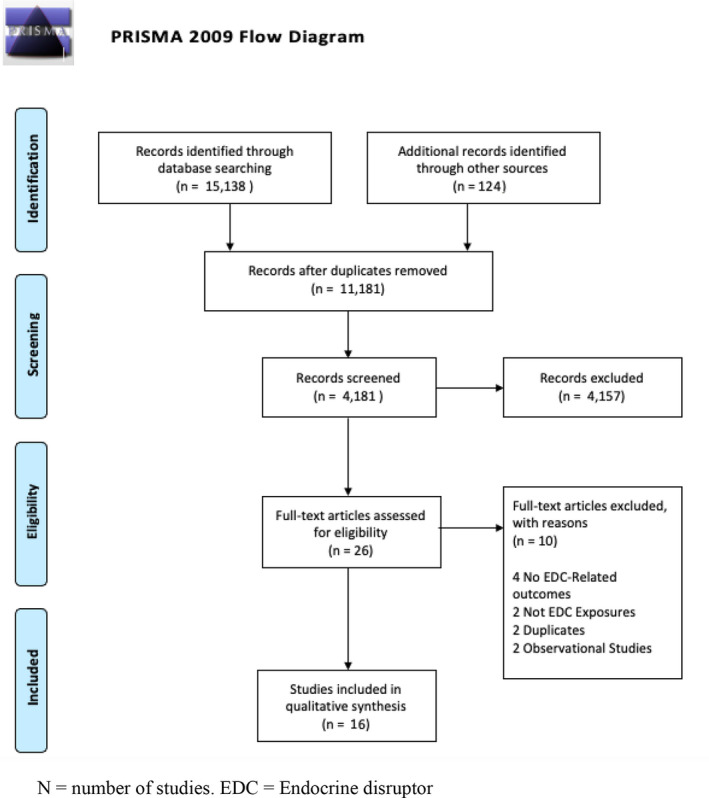
PRISMA flow diagram for article processing during review. EDC, endocrine disruptor; *N*, number of studies

The database searches yielded 15 362 articles. A total of 11 181 duplicates were removed, leaving 4181 articles for title and abstract screening. After title and abstract scanning, 26 articles were considered relevant, and their full manuscripts were reviewed for inclusion. Of these studies, 16 met the inclusion criteria (Figure 1). Ten articles were excluded, with reasons for exclusion also included in Figure 1.

### 3.2 Study characteristics

Key characteristics of each study are presented in Table 1. The studies were published between 2009 and 2021. Population size per study was in the range of 15–355 participants. Three studies included pregnant participants, six included young healthy participants, two included families including parents and children, and two studies examined interventions in solely school‐going children. Four studies included mixed‐gender populations, including one on type 2 diabetic patients, one on cardiac atheromatous disease, and one on men and women aged over 60 years.

**TABLE 1:** Characteristics, interventions, and findings of included studies (*n* = 16)

Study author and year	Study design, population, and study size	Intervention active group	Findings
Carwile, 2009^54^	Non‐RCT Non‐blind College students (*n* = 77)	Impact of stainless‐steel versus poly‐carbonated bottles for drinking beverages on urinary BPA Intervention: Stainless‐steel drinking beverages Control: Poly‐carbonated beverages bottles Duration of intervention: 1 week Summary of findings: Consumption of beverages from poly‐carbonated beverages significantly increased exposure to BPA	Exposure outcome: Geometric mean urinary BPA μg/L Intervention: 1.2 μg/L, 95% CI 1.0–1.4 Control: 2.0 μg/L, 95% CI 1.7–2.4
Hagobian, 2017^55^	Double‐blinded RCT – researchers, lab staff Healthy premenopausal college‐aged women with normal BMI (*n* = 24)	Impact of lifestyle interventions compared to control on urinary BPA concentration. Intervention: Lifestyle interventions include education sessions, BPA‐free tupperware/water bottles, makeup, hygiene and feminine products, organic foods packaged in BPA‐free glass/cardboard containers. Also included the control intervention of weekly newsletter as below Control: Weekly newsletter on BPA information, healthy eating, and physical activity Duration of Intervention: 3 weeks Summary of findings: Lifestyle Intervention significantly reduced BPA urine concentration	Exposure outcome: Urinary BPA ng/ml Intervention: 0.88, 95% CI 0.42–1.34 Control: 1.37, 95% CI 0.59–2.15
Sathyanarayana, 2012^48^	Non‐blinded RCT Families – 10 households with 2 or more children aged 4–8 years, 1 parent living 100% in same household as children (*n* = 40)	Impact of 5‐day complete dietary replacement compared to education on urinary phthalates and BPA Intervention: Dietary replacement consistent of fresh/organic food, catered foods prepared without plastics Control: Education alone, conducted via handouts Duration of intervention: 5 days Summary of findings: Dietary intervention caused unexpectedly statistically significant increase in levels of urinary BPA Caused by food contamination with DEHP – high concentrations of DEHP in ground coriander and milk	Exposure outcome: Urinary BPA μg/L Intervention: 1.6, 95% CI 0.9–2.3 Control: 1.4, 95% CI 0.7–2.1
Rudel, 2011^56^	Non‐randomized non‐blinded crossover trial Five families who frequently consumed canned foods, excluding night shifts, low‐carb diets (*n* = 20)	Impact of EDC‐reducing diet versus typical diet on urinary EDC analytes Intervention: Diet consisting of fresh organic fruit, veg, grains, and meats. Avoiding canned foods, plastic utensils, and non‐stick cookware. Glass containers with BPA‐free plastics, stainless‐steel water bottles and containers Control: Typical diet Duration of treatment: 3 days Summary of findings: Levels of urinary EDC decreased with intervention and increased again with resumption of typical diet	Exposure outcome: Urinary bPA ng/ml, with 95% CI for slope estimate of chance in geometric mean bPA over time period Intervention: 1.2, 95% CI 0.6–1.6 Control: 3.7, 95% CI –1.6 to −0.55
Sessa, 2021^57^	Interventional School‐going children in Italy (*n* = 130)	Impact of plastic‐free canteen food versus normal mealtime habits on concentrations of urinary BPA. Intervention: Certified compostable materials only. Control: Typical plastic beverage bottles, plates, etc. Duration of intervention: 5 days Summary of findings: Significant reduction in levels of urinary BPA with non‐plastic intervention	Exposure outcome: Urinary BPA ng/ml Intervention: 1.12, 95% CI 0.9–2.21 Control: 1.21, 95% CI 1.2–1.38
Carwile, 2011^58^	Randomized single‐blinded crossover trial University student and staff volunteers (*n* = 75)	Effect of canned soup versus fresh soup on urinary BPA Intervention: Fresh soup Control: Canned soup Duration of intervention: 5 days of each intervention/control, with 2‐day washout period between crossover Summary of findings: Canned soup had 1221% higher urinary BPA compared to fresh soup	Exposure outcome: Urinary BPA μg/L Intervention: 1.1, 95% CI 0.9–1.4 Control: 20.8, 95% CI 17.9–24.1
Bae, 2014^59^	Double‐blinded randomized crossover trial – data analyzers not blinded People aged >60 years (*n* = 120)	Impact of canned containers versus glass containers for drinking beverages on urinary BPA Intervention: Glass containers for beverages Control: Canned containers Duration of intervention: 1 week on each intervention, with weeklong washout period between Summary of findings: Canned beverage avoidance reduced urinary BPA. But this did not translate to improvements in blood pressure	Clinical outcome: BP intervention Control: Exposure outcome: Mean urinary BPA μg/L, ±SD Intervention: 1.13 ± 1.76 Control: 7.93 ± 6.01
Curl, 2019^60^	Single‐blinded RCT Pregnant women recruited in first trimester (*n* = 24)	Impact of organic versus conventional fruit and vegetable consumption on urinary phthalates (PBA, PNP, and TCPY) Intervention: Organic fruit and veg Control: Conventional fruit and veg Duration of treatment: 24 weeks, from second through to third trimester of pregnancy Summary of findings: Organic diet significantly reduces BPA. No difference in PBP or TCPY	Exposure outcome: Urinary 3‐BPA μg/L at 50th centile Intervention: 0.27 μg/L Control: 0.95ug/L
Bethune^61^	RCT – 3 groups CAD patients (*n* = 50)	Consumption of salmon fed with fish oil versus fish oil/rapeseed oil versus rapeseed oil alone on plasma dioxins/DLPCBs, PCBS, flame retardants (PBDEs) Intervention: Salmon fed with rapeseed oil Control: Salmon fed with FO or FO/RO combination Duration of treatment: 6 weeks Summary of findings: Fish with non‐marine feeds had lower contamination levels but higher plasma levels for patients	Exposure outcome: Plasma PCBs, PBDEs DLPCBs (mean ± SD) *PCBs* μg/g^−1^ Intervention: 3.00 ± 0.81 Control: FO 7.25 ± 1.34, FO/RO 4.85 ± 0.64 *Dioxins and DLPCBs pg g* ^*−1*^ Intervention: 435.85 ± 89.03 Control: FO 1432 ± 216 FO/RO 766 ± 323 *Flame retardants PBDEs pg g* ^*−1*^ Intervention: 1047 ± 193 Control: FO 2144 ± 327, FO/RO 1698 ± 243
Sears, 2020^62^	Double‐blinded RCT – researchers, lab staff Pregnant women recruited from antenatal clinics (*n* = 355)	Impact of paint stabilization and dust removal compared to reducing injury hazards on urinary phthalates Intervention: Reducing injury hazards included injury prevention devices, stair gates, cabinet locks, smoke detectors Control: Duration of treatment: Recurrent measure implementation from 32 weeks of gestation through to 3 years of life Summary of findings: Paint non‐randomized & dust removal was associated with lower urinary DEPH/MCOP/MCNP but not u‐MEP	Exposure outcome: Mean urinary DEHP ng/ml Intervention: 73, 95% CI –35 to −5 Control: 91, 95% CI –18 to 19
Guo, 2016^63^	Prospective experimental pilot study Non‐randomized Non‐blinded Crossover trial Healthy female individuals (*n* = 15)	Impact of vitamin C versus no vitamin C on plasma levels of EDC metabolites (18 PCBs, 7 OCPs, and 5 PBDEs Intervention: Vitamin C 1000 mg/day Control samples: Before vitamin C supplementation Duration of treatment: 2 months Outcomes were compared from before and after intervention Summary of findings: Vit C reduced PCBs and OCPs but not PBDEs	Exposure outcome: Plasma PCBs and PBDEs *Mean of sum PCBs x 9, ng/mL ± SE* Intervention: 0.48 ± 0.09 Control: 0.52 ± 0.10 Mean of sum PBDEs ng/mL Intervention: 1.04 ± 0.34 Control: 1.08 ± 0.38
Dusanov, 2019^64^	Non‐blinded RCT Men and women age 35–70 years (*n* = 133)	Impact of fatty fish (salmon) consumption versus nuts on serum POPs Intervention: Consumption of fatty fish and nuts Control: Usual diet with avoidance of fatty fish/nuts Duration of treatment: 6 months Summary of findings: No reduction in POPs with fatty fish consumption	Exposure outcome: 5× Organochlorinated compounds (pg/ml) 2× Dioxin‐like PCBs (pg/ml) 8× non‐dioxin‐like PCBs
Kahleova, 2016^65^	Non‐blinded RCT Men and women with T2DM (*n* = 74)	Intervention: Vegetarian diet (no fish or meat) Control: Isocaloric conventional antidiabetic diet Duration of treatment: 12‐week intervention period Summary of findings: No reduction of serum levels w vegetarian versus conventional diet. Reduction in serum level POPs with reduction in HbA1c – independent of BMI	Exposure outcome: Serum levels of POPs *Sum PCBs ng/g* Intervention: 4.83, 95% CI 4.58–5.1 Control: 4.16, 95% CI 3.94–4.37
Park, 2021^66^	Prospective non‐controlled experimental trial Blinding of data analyzers to results Female college students (*n* = 30)	Impact of reducing consumption of fast/processed food on urinary BPAs and menstrual pain Intervention: Small group education (90‐min session on EDC information and impact on women’s reproductive health problems), sources of EDCs in food, cooking and containers, strategies to reduce BPA exposure in dietary habits, follow‐up monitoring, peer‐support via social network communication Control: Baseline period before intervention Duration of treatment: Single intervention Summary of findings: Intervention reduced pain for 3 cycles and reduced urinary BPAs for 2 cycles	Clinical outcome: Difference in menstrual pain scores between low and high adherence groups Intervention: 6 (1–8) Control: 8 (6–10) Exposure outcome: Urinary BPA levels μg/gCr Intervention: 0.41 (0.06–1.42) Control: 0.99 (0.22–3.99)
Brucker‐Davis, 2015^67^	Prospective non‐randomized trial Pregnant women (86 enrolled) – 44 in long‐term neuropsychiatric follow‐up of neonate (*n* = 44)	Impact of iodine supplementation versus control on neurodevelopmental outcomes and maternal milk EDCs. Intervention: Iodine supplementation of 150 μg/day in iodine‐enriched pregnancy vitamins. Control: Pregnancy vitamins not enriched with iodine Duration of treatment: From first trimester through pregnancy Summary of findings: Exposure to PCB118 linked with dysfunctional early language development – not improved with iodine supplementation	Exposure outcome: 15 various EDCs
Harley, 2016^68^	Prospective non‐randomized experimental study Latina girls	Impact of choosing personal care products free of phthalates, parabens, and phenol Intervention: Consumer‐choice of specific EDC‐free personal care products as specified on product label Control: Pre‐intervention measurement Duration of treatment: 3‐day intervention period Summary of findings: Intervention was associated with 27.4% reduction in urinary mono‐ethyl phthalate levels, 43.9% reduction in methyl parabens, and 45.5% reduction in propyl parabens. No changes in mono‐n‐butyl phthalate or mono‐isobutyl phthalates	Exposure outcomes: Gravity‐corrected conc (SE), ng/ml, with 95% CI percent change *MonoEthylPhthalate (MEP)* Intervention: 56.4 (1.1) Control: 78 (1.1) Percent change: −27.4%, 95% CI –39.3 to −13.2 *Mono‐n‐butyl phthalate (MnBP)* Intervention: 25.1 (1.1) Control: 28.3 (1.1) Percent change: −11.3%, 95% CI –22.2 to 1.1 *Mono‐isobutyl phthalate (MiBP)* Intervention: 15.2 (2.3) Control: 15.2 (1.1) Percent change: −0.5%, 95% CI –12.6 to 13.3 *Methyl Paraben* Intervention: 43.2 (1.2) Control: 77.4 (1.2) Percent change: −43.9%, 95% CI –61.3 to −18.8 *Propyl Paraben* Intervention: 12.3 (1.2) Control: 22.6 (1.3) Percent change: −45.4%, 95% CI –63.7 to −17.9
Lu, 2006^69^	Prospective non‐randomized experimental study Elementary‐level children	Impact of food replacement with organic substitutes on exposure to EDCs Intervention: Organic food diet Control: Conventional diet Duration of treatment: 5 days Summary of findings: Organic diet caused immediate reduction in median urinary concentrations of organophosphorus pesticide metabolites	Exposure outcome: Urinary metabolites (mean ± SD) (ug/L) *MDA* Intervention: 0.3 ± 0.9 Control: 2.9 ± 50 *TCPY* Intervention: 1.7 ± 2.7 Control: 7.2 ± 5.8 *IMPY* Intervention: <0.7 ± 0.1 Control: <0.7 ± 0.2 *DEAMPY* Intervention: <0.2 ± 0.1 Control: 0.37 ± 2.2 *CMHC* Intervention: <0.2 ± 0.03 Control: <0.2 ± 003

Abbreviations: BMI, body mass index; BPA, Bisphenol A; DEPH, sum of molar mass of mono‐2‐ethyl‐5‐hydroxyhexyl, mono‐2‐ethyl‐5‐oxohexyl phthalate and mono‐2‐ethyl‐5‐carboxypentyl phthalate; DLCPB, dioxin‐like polychlorinated biphenyls; FO, fish oil; MCNP, mono‐carboxynonyl phthalate; MCOP, mono‐carboxyoctyl phthalate; MEP, mono‐ethyl phthalate; PBA, 3‐Phenoxybenxoic; PCB, polychlorinated biphenyl; PNB, propylene glycol n‐Butyl ether; POP, persistent organic pollutants; RCT, randomized controlled trial; RO, rapeseed oil; T2DM, type 2 diabetes mellitus; TCPY, 3,5,6‐trichloro‐2pyridinol.

### 3.3 Risk of bias in studies

Of the 16 included studies, eight were randomized controlled trials. In addition, there were three crossover trials and six non‐randomized experimental trials. The risk of bias assessment is presented in Table 2.

**TABLE 2:** Risk of bias assessment of included studies (*n* = 16)

			Bias from randomization process	Bias due to deviations from intended interventions	Missing outcome data	Risk of bias in measurement of the outcome	Bias in selection of reported results
*RCTs* ^a^							
Sears, 2020			M	L	L	L	L
Curl, 2019			L	L	L	M	M
Dusanov, 2019			L	M	M	L	L
Hagobian, 2018			L	L	L	L	L
Brucker‐Davis, 2015			L	M	M	L	L
Sathyanarayana, 2012			M	M	L	L	L
Bethune, 2006			M	M	L	L	L
*Crossover RCTs* ^b^							
Bae, 2014			L	L	L	L	L
Carwile, 2011			L	M	L	L	L
Rudel, 2011			M	L	M	L	L
	**Bias due to confounding**	**Bias in selection of participants into the study**	**Bias in classification of Interventions**	**Bias due to deviations from intended interventions**	**Bias due to missing Data**	**Bias in measurement of outcomes**	**Bias in selection of reported results**
*Non‐randomized interventional trials* ^c^							
Park, 2021	M	L	L	L	L	M	M
Sessa, 2021	L	L	L	L	L	L	L
Guo, 2016	M	S	L	L	L	M	M
Carwile, 2009	L	L	L	L	L	L	M
Harley, 2016	M	L	L	L	L	M	L
Lu, 2006	L	M	L	L	L	L	M

Abbreviations: L, low risk of bias; M, some concern of bias; RCT, randomized controlled trial; S, serious risk of bias.

^a^ ROB2 Tool for RCTs.

^b^ ROB2 Tool for crossover trials.

^c^ ROBINS‐I for non‐randomized interventional trials.

## 4 DISCUSSION

The present systematic review found high‐quality evidence to support the consumption of organic food, avoidance of plastics, and avoidance of canned food and beverages in reducing dietary exposure to endocrine disruptors. Interventions including avoidance of fast food, iodine supplementation, vegetarian diet, fatty fish diet, alteration of personal care products, removal of dust, and altering fish feed are all supported by high‐quality evidence. Avoidance of plastics in the diet was incorporated into interventions by using glass or stainless‐steel bottles and containers, and cardboard rather than plastic packaging (or no packaging at all), and avoidance of plastic utensils and non‐stick pans in the kitchen. These interventions yielded significant reductions in exposure to EDCs, measured as urinary BPAs or other EDC metabolites. Minimizing consumption of canned food and beverages also significantly reduces dietary exposure to EDCs. For women and families, this is particularly useful in the preparation of food and choices of baby food. There is observational evidence to support avoidance of bottle feeding and processed baby food to effectively reduce exposure to EDCs.^34^, ^35^ Avoiding canned food and processed food is an effective way to minimize dietary exposure to EDCs.

### 4.1 Replacement of folic acid and iodine

EDC‐related thyroid dysfunction has been linked to neurodevelopmental and cognitive dysfunction.^36^, ^37^, ^38^ Iodine deficiency itself has been shown to increase the risk of attention‐deficit/hyperactivity disorder (ADHD) and low IQ.^39^, ^40^ Although there is ample observational evidence that iodine supplementation reduces this risk, this has not yet been demonstrated in prospected experimental trials. While there is observational evidence that the replacement of folic acid is protective against the adverse effects of EDCs on prenatal development^41^ including autistic spectrum disorder,^42^, ^43^ the present review highlights that folic acid as a potential therapy for EDC‐related adverse effects has not yet been investigated in experimental studies. With the prevalence of autistic spectrum disorder rising, particularly in the past 20 years,^44^, ^45^, ^46^, ^47^ this protective potential of folic acid supplementation is an exciting potential protective therapy. The abundance of observational data with the absence of interventional evidence on both folic acid and iodine therapy poses a challenge for academic and clinical communities. The question centers on whether prospective interventional evidence is required before the recommendation of these therapies or, given their low side effect profile and other benefits on prenatal development, whether they can be recommended based on existing observational evidence alone.

### 4.2 Strengths and weaknesses

The strengths of the present study are the comprehensive literature searches including all relevant publications, including non‐English articles with bibliographies also searched for relevant articles, a broad inclusion criteria, and search strategy that allowed all published nutritional interventions to be captured in the review. Heterogeneity of study populations, interventions, and reported outcomes of included studies limit the results analysis. Where a meta‐analysis could be performed on studies that reported urinary BPAs, this would exclude almost half of the studies (*n* = 7/16) that did not report urinary BPA and would significantly narrow the range of interventions included in the present review. Most included studies did not examine clinical outcomes; rather they reported levels of exposure to EDCs. This represents an additional caveat for prospective trial protocols. Investigation may require a two‐step approach, with the first simply being a reduction in body burden, and the second being a change in clinical outcomes. Observational and epidemiological evidence was excluded from the present review. However, epidemiological data are important contributory resources in this discussion. A broader review of epidemiological evidence on interventions to ameliorate the impact of endocrine disruptors is needed as the next step from the present review, with techniques such as the Navigation Guide highly suitable for this purpose. While non‐human studies have informed the pathophysiology of endocrine disruptors, animal studies were not included in this review. Human‐only studies were examined to highlight proven interventions and interpret these for patients, clinicians, and governmental bodies alike.

### 4.3 Interpretation/implications

The present review provides summarized evidence for patients, physicians, and regulatory bodies on strategies to reduce dietary exposure to EDCs. Parents play a pivotal role in influencing what they and their children are exposed to and can take simple yet effective steps to minimize exposure to EDCs. Avoiding plastic‐contained or canned foods and beverages is effective at reducing exposure to EDCs. This can be achieved through choosing non‐packaged foods or cardboard packaging when unavoidable and using glass or stainless‐steel food containers or drinks bottles, rather than plastic alternatives. For infants and young children, avoiding processed foods such as bottle milk and purees is a simple way to reduce EDCs. Breastfeeding and later using pureed fresh foods for children are effective at reducing exposure to endocrine disruptors. The consumption of organic food and avoiding fast or processed foods are recommended. Care should be taken to minimize contamination by plastic and pesticides by choosing foods that are not wrapped in packaging and washing them thoroughly before consumption. However, it should be noted that intervening on EDCs at the individual level puts the burden on parents and their families to identify and reduce exposure, which has been shown to be difficult in many cases to both identify all relevant sources of exposure, in part due to the lack of requirements to disclose the use of chemicals in products.^48^, ^49^ Additionally, many sources of exposure to EDCs are beyond the control of the individual.^48^, ^49^ In high‐income countries, individual interventions are more achievable where consumers are in a position to make informed food choices. This is often not a luxury shared by populations in low‐ and middle‐income countries, where education, access, and resources will limit food choices. Given all these considerations, public and regulatory government policies appropriately put responsibility and ownership on food and production industries across the world to ensure a healthy and safe supply of food.^49^

Healthcare providers should be aware that endocrine disruptors propose significant risk to reproductive health and prenatal development.^49^ Recommendations from professional bodies may include those such as from the American College of Obstetricians and Gynecologists,^50^ centered around the environmental health history with counseling for a reduction in exposure. Education among medical communities on this issue is essential, incorporating the interventions highlighted here as effective strategies for a reduction in exposure, with integration of environmental health into medical training.^50^ There is also an urgent need for further research into effective interventions to reduce exposure to EDCs or ameliorate its adverse effects. While the present review did find 14 studies, just three of them were of high quality, underling the need for further investigation. This is a novel and exciting area, and one that promises investigators a huge opportunity to improve human health globally.

Finally, this review is a call to action for government and regulatory bodies. It is known endocrine disruptors have a significant cost to the health of women, families, and children at every stage of development and early life, as well as placing significant demand on gynecological and women’s health services.^51^ In addition, the neurobehavioral morbidity of EDCs contributes significant challenges to individual and family well‐being and to healthcare costs.^52^, ^53^ Policy and regulatory strategies should require and incentivize minimizing food packaging, avoiding plastic packaging where possible, and encouraging consumption of fresh and organic food. All of these are proven to effectively reduce exposure to endocrine disruptors, with all the health and economic benefits this entails. Further, these interventions have huge benefits in terms of environmental health and waste reduction. Government interest in the minimization of endocrine disruptors is an extremely worthwhile endeavor, with great benefits to be gleaned from simple yet effective strategies outlined in the present review.

## 5 CONCLUSION

Addressing the effects of EDCs and minimizing exposure to chemicals is an issue requiring urgent attention. While government and regulatory bodies are working on policy solutions, individual‐level interventions can help reduce exposure. While intervention in human studies is still in its early stages, there are effective nutritional interventions that may reduce the adverse effects of EDCs. The avoidance of plastic containers, bottles, and packaging, and canned food is shown to significantly reduce exposure to endocrine disruptors. Consumption of fresh and organic food is also effective at reducing exposure, as is prevention of dust accumulation around the home. While regulatory and further high‐quality experimental research is required into therapies such as anti‐oxidant therapy and iodine and folic acid replacement, the present review presents effective and helpful strategies to reduce nutritional exposure to endocrine disruptors.

## CONFLICT OF INTEREST

JC is the current President of the International Federation of Gynecology and Obstetrics (FIGO) and sits on the board of directors for two non‐profit organizations (the Forum Institute and Heartland Health Alliance). She has no financial conflicts of interest to disclose. The other authors have no conflicts of interest.

## AUTHOR CONTRIBUTIONS

The study concept was conceived by FMMcA. The scope of the study was guided by FMMcA, LG, JC, TJW, Mh, MH, and AMC. The study was designed with input by all authors. The search was conducted by GAC and SL. Risk of bias assessment was performed by GAC and SL. The manuscript was first drafted by GAC and then critically appraised by all authors. All authors approved the final version of this manuscript before submission. The corresponding author declares that all listed authors meet criteria for authorship, having been significant contributors to this project for its entire duration.

## DATA AVAILABILITY STATEMENT

Data sharing is not applicable to this article as no new data were created or analyzed in this study.
